# Histone variants: The bricks that fit differently

**DOI:** 10.1016/j.jbc.2024.108048

**Published:** 2024-12-04

**Authors:** Youssef A. Hegazy, Hejer Dhahri, Nour El Osmani, Smitha George, Darrell P. Chandler, Yvonne N. Fondufe-Mittendorf

**Affiliations:** 1 Department of Epigenetics, Van Andel Research Institute, Grand Rapids, Michigan, USA; 2 Department of Molecular and Cellular Biochemistry, University of Kentucky, Lexington, Kentucky, USA

**Keywords:** histone variants, nucleosome structure, core histones, chromatin accessibility, chromatin dynamics, gene regulation

## Abstract

Histone proteins organize nuclear DNA in eukaryotic cells and play crucial roles in regulating chromatin structure and function. Histone variants are produced by distinct histone genes and are produced independently of their canonical counterparts throughout the cell cycle. Even though histone variants may differ by only one or a few amino acids relative to their canonical counterparts, these minor variations can profoundly alter chromatin structure, accessibility, dynamics, and gene expression. Histone variants often interact with dedicated chaperones and remodelers and can have unique post-translational modifications that shape unique gene expression landscapes. Histone variants also play essential roles in DNA replication, damage repair, and histone–protamine transition during spermatogenesis. Importantly, aberrant histone variant expression and DNA mutations in histone variants are linked to various human diseases, including cancer, developmental disorders, and neurodegenerative diseases. In this review, we explore how core histone variants impact nucleosome structure and DNA accessibility, the significance of variant-specific post-translational modifications, how variant-specific chaperones and remodelers contribute to a regulatory network governing chromatin behavior, and discuss current knowledge about the association of histone variants with human diseases.

Eukaryotic genomes are packaged into chromatin, a complex structure of repeating nucleosomes containing ∼147 bp of DNA wrapped around a histone octamer (1, 2, 3, 4). Because histone proteins are rich in lysine and arginine, they carry a net positive charge, enabling histones to interact tightly with the negatively charged DNA phosphate backbone (4, 5). The histone octamer contains two copies of each core histone (H2A, H2B, H3, and H4), forming the nucleosome by assembling an (H3–H4)_2_ tetramer with H2A–H2B dimers (Fig. 1*A*) (4, 6). To create a stable octamer scaffold, these histone proteins are connected *via* a docking domain located in the H2A C-terminal region (4). While all core histones have an N-terminal tail that protrudes from the nucleosome, H2A has an N-terminal tail and a C-terminal tail. Thus, a canonical nucleosome has 10 flexible tails extending from the nucleosome core particle (Fig. 1*A*) (4). The linker DNA between nucleosomes is bound by the linker histone H1, which stabilizes the higher-order chromatin structure (4, 6).

**Figure 1:**
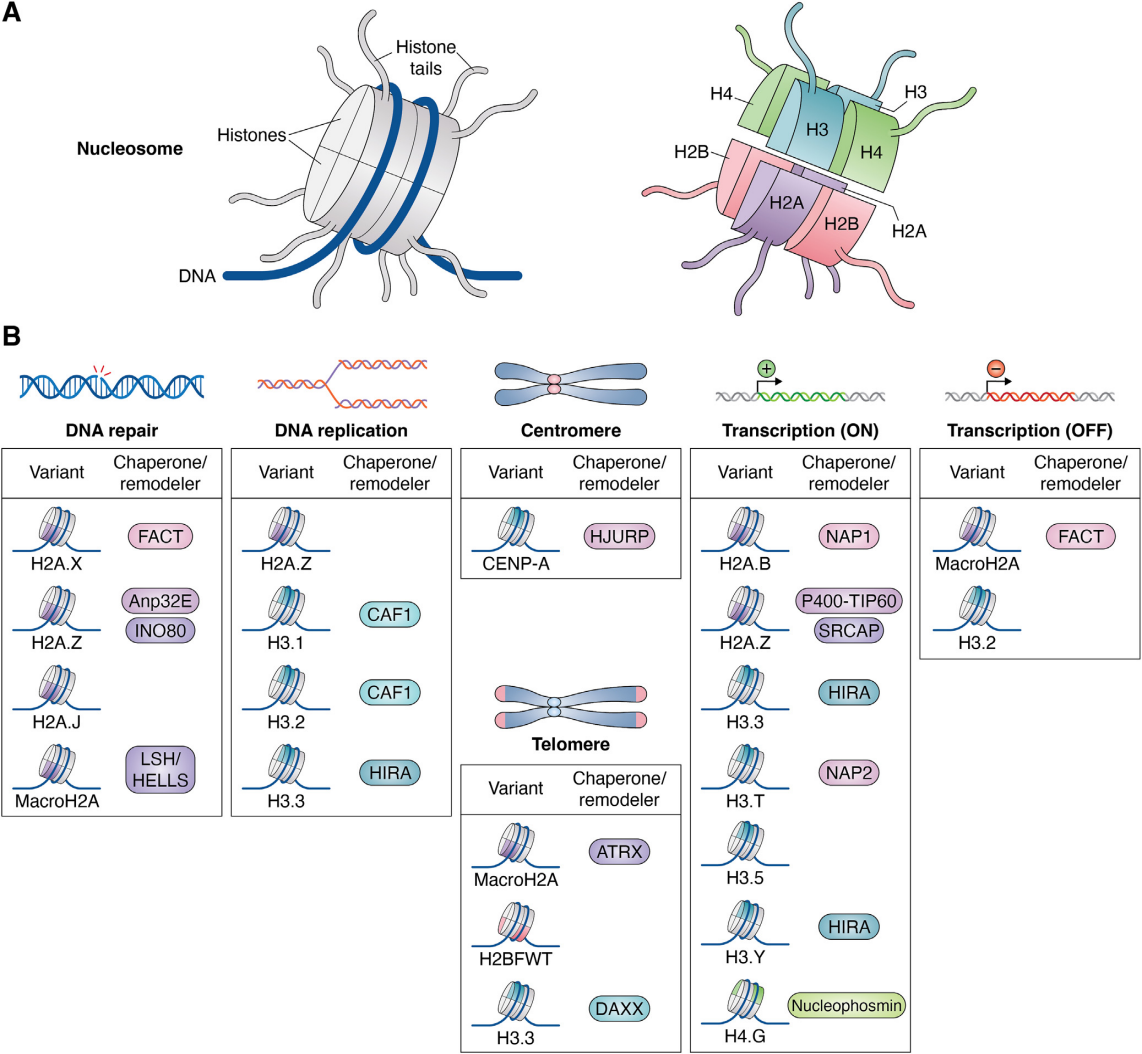
**Summary of the roles of core histone variants in biological processes.***A*, a schematic representing the components of nucleosome core particle (NCP). *B*, an illustration of core histone variant categorization based on their involvement in diverse biological processes in human cells. Core histone variants are involved in DNA repair, replication, and transcriptional regulation, either activation or repression. They are also associated with centromeres and telomeres. The names of relevant chaperones/chromatin remodelers associated with each variant are also indicated.

Canonical histones are expressed during the S phase of the cell cycle and are incorporated into chromatin during DNA replication (7, 8). During DNA replication, histones get disrupted by the replication fork (6, 9). Newly synthesized histones and pre-existing disrupted histones must be incorporated into newly replicated chromatin (9). These replication-coupled histones are, therefore, often referred to as “canonical” histones, in contrast to histone variants that are usually incorporated into chromatin throughout the cell cycle and at specific times and in specific cells or tissues to enact specific gene expression patterns (10, 11). Multiple genes usually express the replication-coupled histones, and their mRNAs lack introns and polyadenylated tails but possess a 3′ stem–loop structure instead (12, 13). The 3′ stem–loop structure in the histone mRNAs is crucial for their replication dependency. They are bound by the stem–loop binding protein, whose expression is also cell cycle dependent, increasing in the S phase to facilitate the processing of the canonical histone mRNA transcripts and finally degrading at the end of the S phase to prevent further accumulation of canonical histone mRNA transcripts (12, 14, 15, 16). Some replication-coupled histone genes can produce polyadenylated mRNA, especially in terminally differentiated cells that no longer divide (17).

Each histone family consists of various paralogs, which may differ from their canonical counterparts by a few to many amino acids and even domain changes. Those paralogs are called histone variants when they usually meet specific criteria: exhibit cell cycle–independent expression, are encoded by a single gene, and have mRNAs with introns and polyadenylated tails (11, 18). However, these criteria are still controversial, and it can sometimes be challenging to determine whether a particular histone is a “variant.” This complexity possibly arises because specific histones can exhibit cell type–specific and condition-specific expression levels. In addition, some transcripts switch from having a 3′ stem–loop structure to being polyadenylated under certain conditions (17, 19, 20). In general, incorporating histones into chromatin requires “chaperones” that escort histones to their target regions and chromatin remodelers that reposition nucleosomes along the DNA strand, eject pre-existing nucleosomes from chromatin, insert new nucleosomes, or replace canonical histones with histone variants (21, 22, 23).

In humans, many histone genes occur in well-defined clusters, whereas others are dispersed across the genome (11). For instance, the HIST1 cluster on chromosome six contains about 60 histone genes, and the HIST2 and HIST3 clusters on chromosome one contain 14 and four genes, respectively. The human genome comprises several histone genes, including genes encoding canonical histones and variant histones, and some pseudogenes (11), where some “previously annotated” pseudogenes were found later to be expressed, producing functional proteins (11, 24). As mentioned previously, histone variants differ from their canonical counterpart by a few to many amino acids or domain changes. They are sometimes classified into homomorphous and heteromorphous variants (25, 26). Homomorphous histone variants exhibit a few amino acid substitutions scattered throughout the protein (*e.g.*, TH2A and H3.3). In contrast, heteromorphous variants exhibit substantial differences in amino acid sequence and structure (*e.g.*, MacroH2A, H2A.Z, H2A Barr-body-deficient, and CENP-A) (26).

Since all histones affect chromatin organization, small amino acid changes can significantly impact chromatin structure and dynamics and gene expression (27). Histone variants can alter chromatin by modifying histone–histone or histone–DNA interactions and nucleosome stability (28, 29, 30). They undergo unique post-translational modifications (PTMs) (31, 32), forming distinct chromatin complexes that impact gene expression (33, 34, 35, 36). Histone variant expression is highly dynamic with distinctive temporal properties/features (31, 37). Our understanding of histone variants and their influence on chromatin structure, gene expression, and cellular function is continually evolving. This review will delve into the diverse landscape of core histone variants and how they affect chromatin structure, as well as explore their implications and functions in health and disease.

## Histone variants and their role in chromatin structure and dynamics

### H2A variants

**Figure 2:**
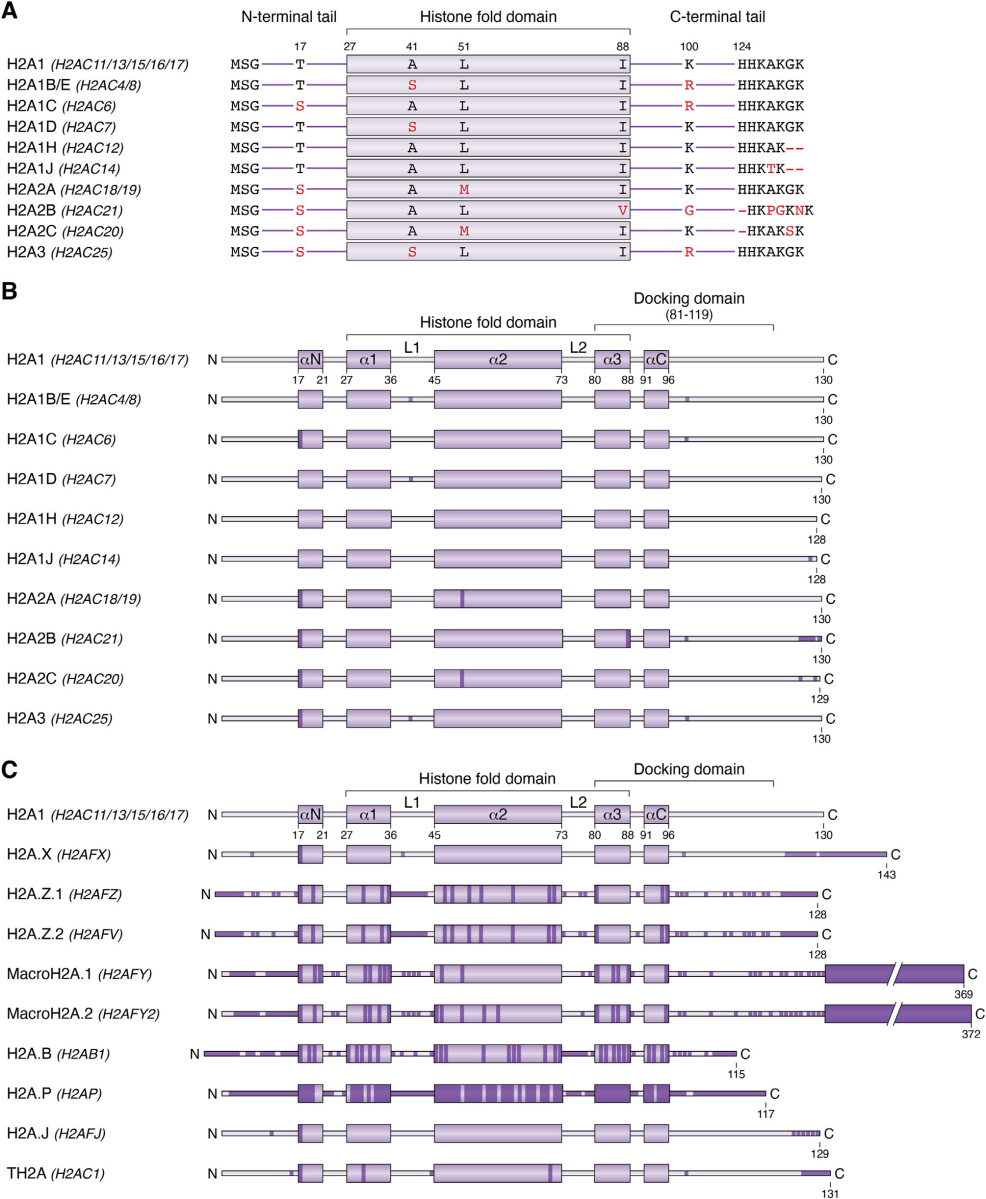
**A schematic representation of amino acid sequence alignment of canonical and variant H2A proteins.***A*, amino acid sequence alignment among canonical histone H2A sequences was performed using the “UniProt Align” tool. The name of the gene(s) encoding each protein is provided between parentheses. The isoform used for the reference sequence is H2A1 (UniProt ID: P0C0S8), encoded by *H2AC11/13/15/16/17* genes. *B*, a schematic representation of the amino acid sequence alignment among canonical histone H2A sequences, with *darker-colored regions* representing substitutions. *C*, a schematic representation of amino acid sequence alignment of histone H2A variants compared with the reference sequence of H2A1 (UniProt ID: P0C0S8), encoded by *H2AC11/13/15/16/17* genes.

The H2A family is the most studied of all variants and has the most extensive repertoire of variants among canonical histones. In humans, 16 genes encode for canonical histone H2A and 11 genes encode H2A histone variants dispersed across different chromosomes (Table 1). H2A variants exhibit a broad spectrum of sequence identity compared with canonical H2A, with some differing in only a few amino acids (*e.g.*, H2A.X, H2A.J) and others displaying as low as ∼18% sequence similarity (*e.g.*, H2A.P) (Fig. 2, *A*–*C*). These changes highlight distinct functional and structural properties of H2A variants, contributing to their specialized roles in chromatin organization, gene regulation, and DNA repair processes (Fig. 1*B*) (25, 38, 39).

**Table 1:** Histone variants and their gene names and genomic locations

Protein name	UniProt ID	Gene name	Genomic location	Expression	Intron containing?
H2A1 (canonical)	P0C0S8	*H2AC11/13/15/16/17*	6q22.2	Replication dependent	No
H2A1B/E (canonical)	P04908	*H2AC4/8*	6q22.2	Replication dependent	No
H2A1C (canonical)	Q93077	*H2AC6*	6q22.2	Replication dependent	Yes
H2A1D (canonical)	P20671	*H2AC7*	6q22.2	Replication dependent	No
H2A1H (canonical)	Q96KK5	*H2AC12*	6q22.2	Replication dependent	No
H2A1J (canonical)	Q99878	*H2AC14*	6q22.2	Replication dependent	No
H2A2A (canonical)	Q6FI13	*H2AC18/19*	1q21.2	Replication dependent	No
H2A2B (canonical)	Q8IUE6	*H2AC21*	1q21.2	Replication dependent	No
H2A2C (canonical)	Q16777	*H2AC20*	1q21.2	Replication dependent	No
H2A3 (canonical)	Q7L7L0	*H2AC25*	1q42.13	Replication dependent	Yes
H2A.X	P16104	*H2AFX*	11q23.3	Replication independent	Yes
H2A.Z.1	P0C0S5	*H2AFZ*	4q23	Replication independent	Yes
H2A.Z.2.1	Q71UI9	*H2AFV*	7p13	Replication independent	Yes
H2A.Z.2.2	Q71UI9	*H2AFV*	7p13	Replication independent	Yes
macroH2A.1.1	O75367	*H2AFY*(*Macroh2a1*)	5q21–q31	Replication independent	Yes
macroH2A.1.2	O75367	*H2AFY*(*Macroh2a1*)	5q21–q31	Replication independent	Yes
macroH2A.2	Q9P0M6	*H2AFY2*(*Macroh2a2*)	10q22.3	Replication independent	Yes
H2A.B1	P0C5Y9	*H2AB1*	Xq28	Replication independent	No
H2A.B2/3	P0C5Z0	*H2AB2/3*	Xq28	Replication independent	No
H2A.P	O75409	*H2AP (HYPM)*	Xp11.4	Replication independent	No
H2A.J	Q9BTM1	*H2AFJ*	12p12.3	Replication independent	Yes
TH2A (H2A1A)	Q96QV6	*H2AC1*	6p21–22	Replication dependent	No
H2B1C/E/F/G/I (canonical)	P62807	*H2BC4/6/7/8/10*	6p22.2	Replication dependent	No
H2BK1	A0A2R8Y619	*H2BK1*	7q36.1	Not known	Yes
H2B.N	P0DW85	*H2BN1*	17q11.2	Not known	Yes
H2BFWT	Q7Z2G1	*H2BW1*	Xq22.2	Replication independent	Yes
H2BFM	P0C1H6	*H2BW2*	Xq22.2	Replication independent	Yes
TSH2B	Q96A08	*H2BC1*	6p22.2	Replication dependent	No
H2B1B	P33778	*H2BC3*	6p22.2	Replication dependent	No
H2B1D	P58876	*H2BC5*	6p22.2	Replication dependent	No
H2B1H	Q93079	*H2BC9*	6p22.2	Replication dependent	No
H2B1J	P06899	*H2BC11*	6p22.2	Replication dependent	
H2B1K	O60814	*H2BC12*	6p22.2	Replication dependent	Yes
H2B1L	Q99880	*H2BC13*	6p22.1	Replication dependent	No
H2B1M	Q99879	*H2BC14*	6p22.1	Replication dependent	No
H2B1N	Q99877	*H2BC15*	6p22.1	Replication dependent	No
H2B1O	P23527	*H2BC17*	6p22.1	Replication dependent	No
H2B2F	Q5QNW6	*H2BC18*	1q21.2	Replication dependent	Yes
H2B2E	Q16778	*H2BC21*	1q21.2	Replication dependent	No
H2B3B	Q8N257	*H2BC26*	1q42.13	Replication dependent	No
H3.1 (canonical)	P68431	*H3C1/2/3/4/6/7/8/10/11/12*	6p22.1-6p22.2	Replication dependent	No
H3.2 (canonical)	Q71DI3	*H3C13/14/15*	1q21.2	Replication dependent	No
H3.3	P84243	*H3-3A/B*	1q42.12|17q25.1	Replication independent	Yes
H3.T	Q16695	*H3-4*	1q42.13	Replication dependent	No
H3.5	Q6NXT2	*H3-5*	12p11.21	Replication independent	No
H3.Y	P0DPK2	*H3Y1*	5p15.1	Replication independent	No
H3.X	P0DPK5	*H3Y2*	5p15.1	Replication independent	No
CENP-A	P49450	*CENPA*	2p23.3	Replication independent	Yes
H4 (canonical)	P62805	*H4C1/2/3/4/5/6/8/9/11/12/13/14/15/16*	6p22.1-6p22.2, 1q21.2, 12p12.3	Replication dependent	No
H4.G (H4.7)	Q99525	*H4C7*	6p22.2	Replication dependent	No

#### H2A.X

The *H2AFX* gene encodes H2A.X, which may form heterotypic or homotypic nucleosomes containing either one or two copies of H2A.X, respectively (40). H2A.X deposition is mediated entirely by general histone chaperones (41, 42, 43). Still, there are no known H2A.X-specific chaperones (44) to date. *H2AFX* generates both poly(A)+ and poly(A)- mRNA isoforms with distinct roles in different cell types. The poly(A)- isoform supports histone deposition in DNA damage response (DDR) regions, whereas the poly(A)+ isoform is crucial for new H2A.X synthesis, essential for effective DDR (45).

H2A.X differs from canonical H2A at one amino acid in the histone-fold domain and displays 13 additional amino acids, including an SQ motif in the C-terminal region (Fig. 2*C*). This SQ motif harbors an S139 residue that is essential for DDR (46, 47). When DNA is damaged, DNA repair kinases immediately phosphorylate H2A.X S139 to form γ-H2A.X, destabilizing the nucleosome by impairing the binding of the linker histone H1 to the DNA entry and exit sites. This triggers a signaling cascade that recruits DNA repair proteins (*e.g.*, poly-ADP-ribose polymerase or poly(ADP-ribose) polymerase 1) to the damaged sites (46, 48, 49). Once repaired, γ-H2A.X is either diluted *via* histone exchange or dephosphorylated *via* phosphatases like PP2A, PP4C, or wildtype p53-induced phosphatase one (Wip1) (41, 50, 51, 52). This early DDR event occurs quickly after double-strand break (DSB) induction. Loss or deficiency of γ-H2A.X compromises genomic stability, reduces DSB repair efficiency, and increases tumor susceptibility and radiosensitivity (53, 54).

Interestingly, a distinct H2A.X variant was identified in the early embryos and eggs of the frog *Xenopus laevis* and was named H2A.X-F (55). It carries a C-terminal “SQEF” instead of “SQEY” in the H2A.X, among other substitutions. *In vitro* reconstitution studies showed that this embryonic variant H2A.X-F is essential for mitotic chromatid assembly into chromosomes (56).

#### H2A.Z

H2A.Z is a heteromorphous variant that only shares 60% similarity with the protein sequence of canonical H2A (Fig. 2*C*). There are two H2A.Z subtypes (H2A.Z.1 and H2A.Z.2) encoded by *H2AFZ* and *H2AFV* genes, respectively (25, 39, 57). The two H2A.Z isoforms differ at three amino acid residues, whereas the splice variant H2A.Z.2.2 shows a truncation and some variations in its C-terminal domain (*i.e.*, shorter C-terminal region with changes in six amino acids) (58).

All H2A.Z isoforms regulate gene expression, DNA replication, and chromosome segregation (reviewed in Ref. (59)), although H2A.Z isoform expression is tissue specific, and their functions are nonredundant (25, 59). While histone variant composition significantly affects nucleosome stability, the precise impact of H2A.Z variants remains unclear. Horikoshi *et al.* (60)found that nucleosomes with two H2A.Z copies are less thermally stable than those with one. H2A.Z nucleosomes also show reduced stability compared with canonical H2A, likely because of structural differences in the acid patch and L1 loop (38, 61). Of note is that the N- and C-terminal domains of H2A.Z are involved in internucleosomal spacing and DNA unwrapping, respectively (62). However, other studies indicate that H2A.Z-containing nucleosomes are more stable than canonical H2A-containing nucleosomes (63). This could be attributed, at least partially, to the PTMs on H2A.Z. On the other hand, these differential results could also be due to H2A.Z co-occupancy with other histone variants and different genomic locations of the H2A.Z-containing nucleosomes. For instance, nucleosomes containing H2A.Z and H3.3, which are enriched at the gene enhancer and promoter regions, are less stable than those with their canonical counterparts (64).

Interestingly, H2A.Z was reported to play a role in higher-order chromatin folding at constitutive heterochromatin (65). H2A.Z alters the nucleosomal surface by increasing the acidic nature of the acidic patch of the nucleosome, thus facilitating the binding of HP1α, a unique chromatin-binding protein that preferentially binds condensed higher-order chromatin structures (65).

Functionally, the deposition of H2A.Z isoforms has been linked to DNA repair (Fig. 1*B*), although their role in this process is transient. Upon DNA DSBs, the chromatin remodeler p400 replaces H2A with H2A.Z, opening chromatin to repair factors such as Ku70/80 and BRCA1 (66). This temporal deposition of H2A.Z is crucial for loading repressive complexes that block transcription near break sites (39). This dynamic interplay between H2A.Z and chromatin remodelers/histone chaperones remains an active research area.

In addition, H2A.Z maintains chromatin structure and regulates gene expression during mitosis by remaining at transcription start sites (TSSs) of active genes, enabling their rapid silencing and reactivation during cell cycle transitions (67). H2A.Z also regulates genes critical for pluripotency and differentiation through various molecular mechanisms influenced by its modifications, interactions, and genomic location. It plays a key role in chromosome segregation, particularly in maintaining pericentric heterochromatin stability (68) and centromere function during mitosis (69). H2A.Z is associated with heterochromatin through monoubiquitylation (70). Moreover, its isoforms have distinct roles in the cell cycle: H2A.Z.2 regulates G2/M-associated genes, whereas H2A.Z.1 influences the G1/S phase *via* interactions with c-Myc (71).

#### MacroH2A (mH2A)

MacroH2A.1 and macroH2A.2 are encoded by the *H2AFY* and *H2AFY2* genes, respectively (25, 39). MacroH2A histones, the most distinctive H2A variant, display three substantial structural changes relative to their canonical H2A. The N-terminal region shares 64% similarity with the amino acid sequence of canonical H2A, whereas its C-terminal tail is connected to an extranucleosomal domain composed of a disordered H1-like linker region and a macrodomain (Fig. 2*C*). MacroH2A.1.1 and macroH2A.1.2, known splice variants of the *H2AFY* gene, exhibit a difference in 33 amino acid residues in their macrodomain (25, 39). MacroH2A.1.1 appears to have isoform-specific functions in that it binds to ligands like ADP-ribose derivatives, which results in its recruitment and regulation of poly(ADP-ribose) polymerase one–dependent DNA repair pathway (72).

MacroH2A variants stabilize heterochromatin and nuclear structure by recruiting lamin B1 (LMNB1) to chromatin (72, 73). MacroH2A nucleosomes are more stable, are refractory to SWI/SNF remodeling (74, 75), and have stronger intranucleosomal protein–protein and protein–DNA interactions than H2A nucleosomes (76). The macroH2A H1-like linker region compacts DNA flanking the nucleosome entry/exit sites, reducing chromatin accessibility (77). As a result, macroH2A variants act as a critical transcriptional repressor, especially around ribosomal DNA (73) and X-chromosome inactivation sites (reviewed in Ref. (25)).

Interestingly, macroH2A variants co-occupy the same genomic regions that are enriched in the repressive histone H3K27me3 (trimethylation of histone H3 at lysine 27) and H3K9me3 (trimethylation of histone H3 at lysine 9) marks (25, 73). Likewise, macroH2A variants are subject to PTMs, with ubiquitination being essential for variant deposition and maintaining its repressive functions (78, 79). The PTMs on macroH2A are partially conserved amongst its isoforms, which could explain the distinct biological functions among them (39, 80). Thus, macroH2A and its isoforms intricately cooperate with key regulatory proteins to preserve chromatin integrity, yet its precise functional mechanism must be addressed further.

#### H2A.B

The H2A.Barr-body-deficient histone variant (H2A.Bbd or H2A.B) is encoded by three nearly identical genes (*H2AB1*, *H2AB2*, and *H2AB3*) (39, 81, 82). The name is derived from the absence of this variant in the inactivated X-chromosome (also known as Barr bodies, characterized by condensed chromatin). H2A.B shares 48% identity with the amino acid sequence of canonical H2A (Fig. 2*C*); it is missing the canonical H2AC-terminal tail and has six consecutive arginine residues in its N-terminal region. The H2A.B docking domain is also truncated relative to canonical H2A, contributing to distinct transcriptional activation and mRNA processing functions (83, 84, 85). H2A.B is classified as a short H2A (sH2A) variant.

H2A.B-containing nucleosomes exhibit a fast exchange rate and reduced stability relative to nucleosomes containing the canonical H2A histones (84, 85, 86). H2A.B also has a great binding affinity to newly synthesized DNA, particularly during DNA repair or S-phase (87). Interestingly, H2A.B-containing nucleosomes are only wrapped with ∼120 bp of DNA, which creates a “relaxed” or “open” chromatin structure that is highly accessible and conducive to active transcription (83, 88). The unique H2A.B structure also weakens interactions between H2A.B-H1 and H2A.B-H3, further "loosening” histone–DNA interactions (88). Other studies also show that H2A.B occupancy correlates with H4 acetylation marks, suggesting its role in promoting gene expression (81, 82). Consistently, H2A.B naturally lacks an acidic patch, reducing the acidic nature of the nucleosome, an effect that adversely impacts the nucleosome folding into the 30-nm chromatin fiber and transcription repression (89).

Interestingly, the ability of H2A.B to substitute various H2A variants highlights the functional crosstalk among variants. For instance, H2A.B.3 was shown to replace H2A.Z at exon–intron boundaries in spermatogenic cells, thus regulating splicing and driving specific gene expression patterns (90). While histone chaperones and chromatin remodelers usually orchestrate histone exchange, the H2A–H2B dimer can be spontaneously replaced with an H2A.B–H2B dimer without the assistance of any factors (91). SWI/SNF and ACF remodeling complexes have negligible activity at H2A.B-containing nucleosomes, even in the presence of nucleolin (a chaperone that enhances SWI/SNF function) (86, 92). To date, NAP1 is the only known chaperone that can evict H2A.B from a nucleosome (93), and to the best of our knowledge, no PTMs have been reported for H2A.B variants. However, it is worth noting that H2A.B enhances p300-dependent histone acetylation of neighboring histone tails (86), underscoring the regulatory role of H2A.B in the dynamic modulation of the nucleosomal architecture.

#### H2A.P

Like H2A.B, H2A.P belongs to the sH2A variant group. It is encoded by the *H2AP* gene (HYPM), and it shares only 18% similarity with the amino acid sequence of canonical H2A (Fig. 2*C*). H2A.P lacks critical arginine residues, resulting in relatively weak H2A.P–DNA interactions destabilizing the nucleosome and increasing the chromatin accessibility (25). Not much is known about H2A.P and sH2A in general. sH2A variant levels are lineage specific as their expression is predominant in the testis, implying their essential role during spermatogenesis (reviewed in Ref. (94)). Further research is needed to understand the functional role of sH2A variants, particularly H2A.P, in regulating chromatin structure and genome integrity.

#### H2A.J

The *H2AFJ* gene encodes H2A.J (also known as H2A.22), which shares 95% similarity with the amino acid sequence of canonical H2A (Fig. 2*C*). Like H2A.X, the H2A.J C-terminal domain contains an SQ motif that is phosphorylated upon DNA damage (95). Despite its high similarity to canonical H2A, H2A.J activates DNA damage–specific inflammatory pathways, drives senescence, and remodels chromatin organization. On a structural level, the crystal structure of H2A.J-specific residues revealed no changes in the overall nucleosome structure, possibly implying the recruitment of transcription factors that specifically bind to its C-terminal domain (96). Another study showed that H2A.J deposition weakens the interaction between H1 and linker DNA, increasing chromatin accessibility and gene expression (97). Otherwise, further studies are needed to understand how H2A.J regulates chromatin structure, dynamics, and gene expression.

#### TH2A

TH2A (or H2A1A), a homomorphous H2A variant, is encoded by the *H2AC1* gene. TH2A amino acid sequence is highly similar to the canonical H2A, displaying changes in only 10 amino acids (Fig. 2*C*) (25, 46, 98). TH2A is uniquely expressed in germ cells (*e.g.*, oocytes, testis, and zygotes); however, its functions remain poorly understood (reviewed in Ref. (99)). Nucleosomes containing TH2A have a weakened L1–L1 interaction that is probably because of the TH2A-specific amino acid substitutions (100). Interestingly, the functional dimerization of a histone TH2A and TSH2B (an H2B variant) induces pluripotency and mediates genome reprogramming (101). In mice, TH2A ortholog is phosphorylated at T127 residue in sperm cells and then accumulates in pericentromeric regions, suggesting that TH2A may also be involved in chromatin condensation (102).

In summary, each H2A variant introduces unique structural and functional properties to nucleosomes that influence nucleosome stability and DNA accessibility. H2A.X and its phosphorylated form mediate efficient DNA repair. H2A.Z and H2A.B mark and activate gene transcription, whereas macroH2A.B condenses chromatin and represses gene expression (Fig. 1*B*). General chaperones synergistically cooperate with chromatin remodelers to load H2A variants into chromatin. However, more research is needed to identify possible variant-specific chaperones. H2A variant PTMs can also regulate spatiotemporal functions of variants within and among nucleosomes and is now an active area of research (reviewed in Ref. (80)).

### H2B variants

**Figure 3:**
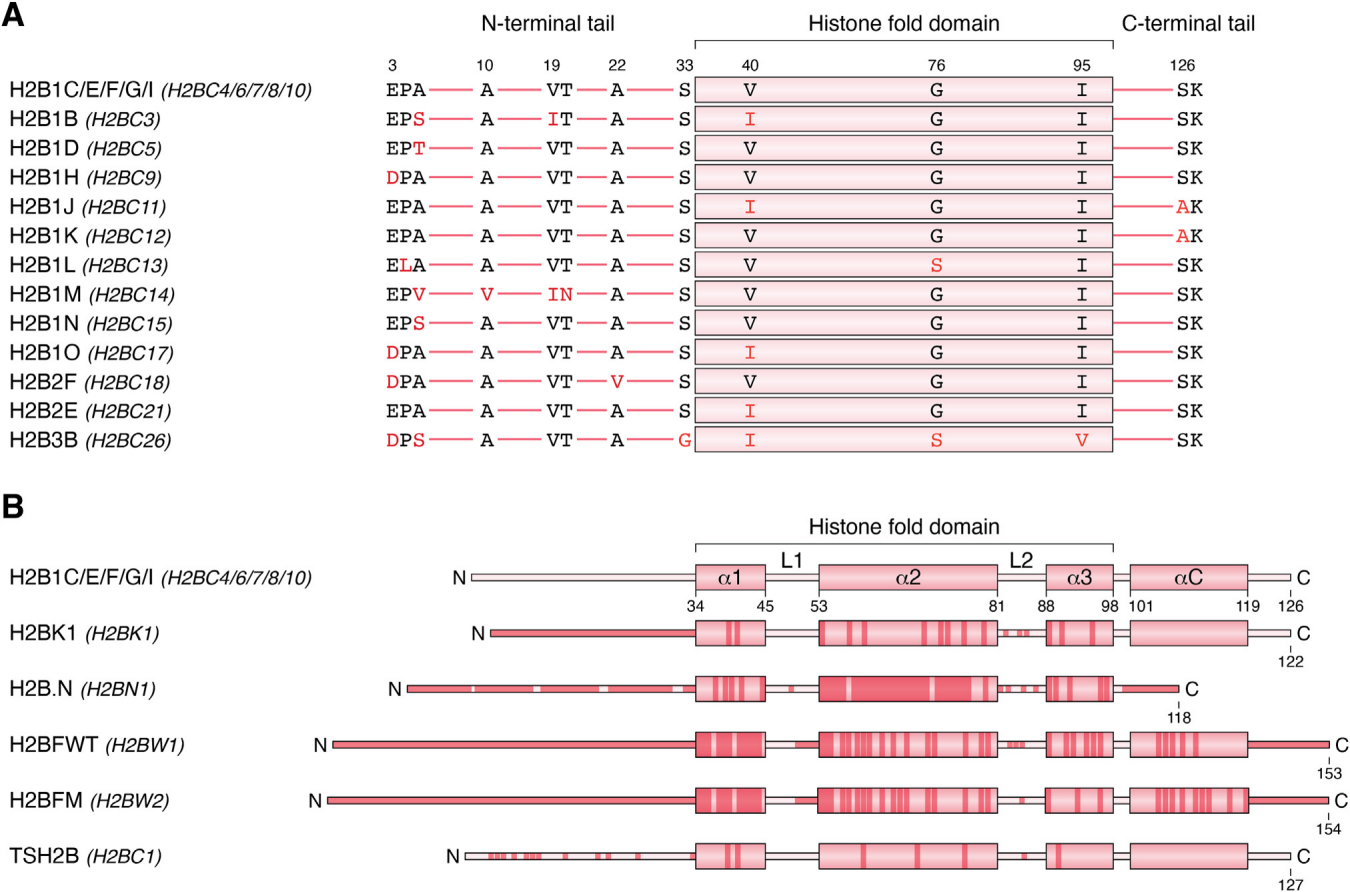
**A schematic representation of amino acid sequence alignment of H2B variants relative to the canonical H2B.***A*, amino acid sequence alignment of histone H2B variants with minor differences compared with the canonical H2B sequence. Sequence alignment was performed using the “UniProt Align” tool. The name of the gene(s) encoding each protein is provided between parentheses. The isoform used for the canonical H2B sequence is H2B1C/E/F/G/I (UniProt ID: P62807), encoded by *H2BC4/6/7/8/10* genes. *B*, a schematic representation of amino acid sequence alignment of histone H2B variants with major differences compared with the canonical H2B sequence (UniProt ID: P62807) encoded by *H2BC4/6/7/8/10* genes. *Darker-color regions* represent substitutions.

More than 25 H2B variant genes/pseudogenes exist in the human genome. Most of them are in histone cluster one on chromosome 6, with the others dispersed across the other chromosomes (Table 1) (11). Because of their high sequence homology, which results in the lack of specific antibodies, H2B variants are the least characterized class of histone variants. For instance, H2B1B/D/H/J/K/L/M/N/O, H2B2F/E, and H2B3B are 12 different H2B variants that have a very few substitutions among each other and relative to the canonical one (Fig. 3*A*). Therefore, there are many open questions about how H2B variants affect chromatin structure and how they function in health and disease.

#### H2BK1

H2BK1 (also known as H2BE1) is encoded by the *H2BK1* gene on chromosome seven (not to be confused with H2B1K encoded by the *H2BC12* gene on chromosome six or with H2B2E encoded by the *H2BC21* gene on chromosome 1). *H2BK1* is one of the intron-containing histone genes. Only 16 amino acid substitutions exist in the H2BK1 histone fold domain (HFD) relative to canonical H2B, but there is a significant divergence in the N-terminal tail (Fig. 3*B*). H2BK1 has an overall lower charge than canonical H2B, which implies that H2BK1-containing nucleosomes should be less compact than a canonical H2B–containing nucleosome (103). The H2BK1 N-terminal tail also contains polyglutamine repeats, which is atypical for H2B proteins and could facilitate protein–protein interactions (104); at the same time, the H2BK1 N-terminal tail is missing key lysine residues that are typically subject to PTMs in canonical H2B. Amino acid substitutions in the second DNA-binding loop (L2) in the H2BK1 HFD could affect DNA binding (103). However, the H2BK1 amino acid substitutions in the H2A- and H4-interacting residues are minor and do not seem to impact H2BK1–H2A or H2BK1–H4 interactions (103).

#### H2B.N

H2B.N is encoded by the *H2BN1* gene on chromosome 17, which is also an intron-containing gene (not to be confused with H2B1N encoded by the *H2BC15* gene on chromosome 6). The H2B.N HFD is very different (only 42.4% identity) than the canonical H2B (Fig. 3*B*). The substitutions include the residues in the H2A- and H4-interacting regions. In addition, H2B.N has a significantly truncated C terminus that is missing essential residues for the nucleosome acidic patch (103, 105, 106), suggesting that H2B.N might confer unique properties to the nucleosome structure or have non-nucleosomal functions (103). Besides, the C-terminal tail of H2B.N is also missing a key amino acid, K120, that usually is subject to ubiquitylation in canonical H2B, which is associated with active transcription, suggesting an impact of the H2B.N C-terminal tail truncation on the nucleosomal structure and gene expression (32, 103, 107, 108).

#### H2B.W

H2BFWT and H2BFM are encoded by *H2BW1* and *H2BW2* on chromosome X, respectively. They can be collectively called H2B.W variants. Both H2B.W variants have ∼50% identity with the canonical H2B HFD, but most of the H2A- and H4-interacting region residues are conserved. H2BFWT and H2BFM have nearly identical extended C-terminal tails, which are longer than the canonical H2B C terminus (Fig. 3*B*). H2B.W variants have significant N-terminal tail divergence between variants and relative to canonical H2B. The highly divergent and significantly longer N- and C-terminal tails of H2B.W variants are expected to significantly impact nucleosome stability, PTM profiles, and gene expression (103), with previous studies indicating that *H2BW1* and *H2BW2* are expressed in human sperm (109, 110, 111, 112, 113).

#### TSH2B

Testis-specific histone H2B (TSH2B, H2BA, or H2B.1) is encoded by the *H2BC1* gene on chromosome six and plays a critical role in the histone–protamine transition during spermatogenesis and after fertilization (27, 114, 115). The TSH2B HFD differs from the canonical H2B HFD at only seven residues (Fig. 3*B*) (29), but most of the residues involved in TSH2B–DNA, –H2A, and –H4 interactions are mostly conserved (103). The crystal structure of the TSH2B-containing nucleosome revealed that S85 (corresponding to N84 in canonical H2B) does not interact with H4 in the nucleosome, unlike N84 (which forms hydrogen bonds with H4 R78) (29). This structural difference could impact nucleosome stability, and a nucleosome reconstitution experiment showed that TSH2B reduced nucleosome stability relative to nucleosomes containing canonical H2B (116). The TSH2B N-terminal region has 12 amino acid substitutions relative to canonical H2B, including some amino acids (like S/T) that can become phosphorylated (103, 116, 117).

### H3 variants

**Figure 4:**
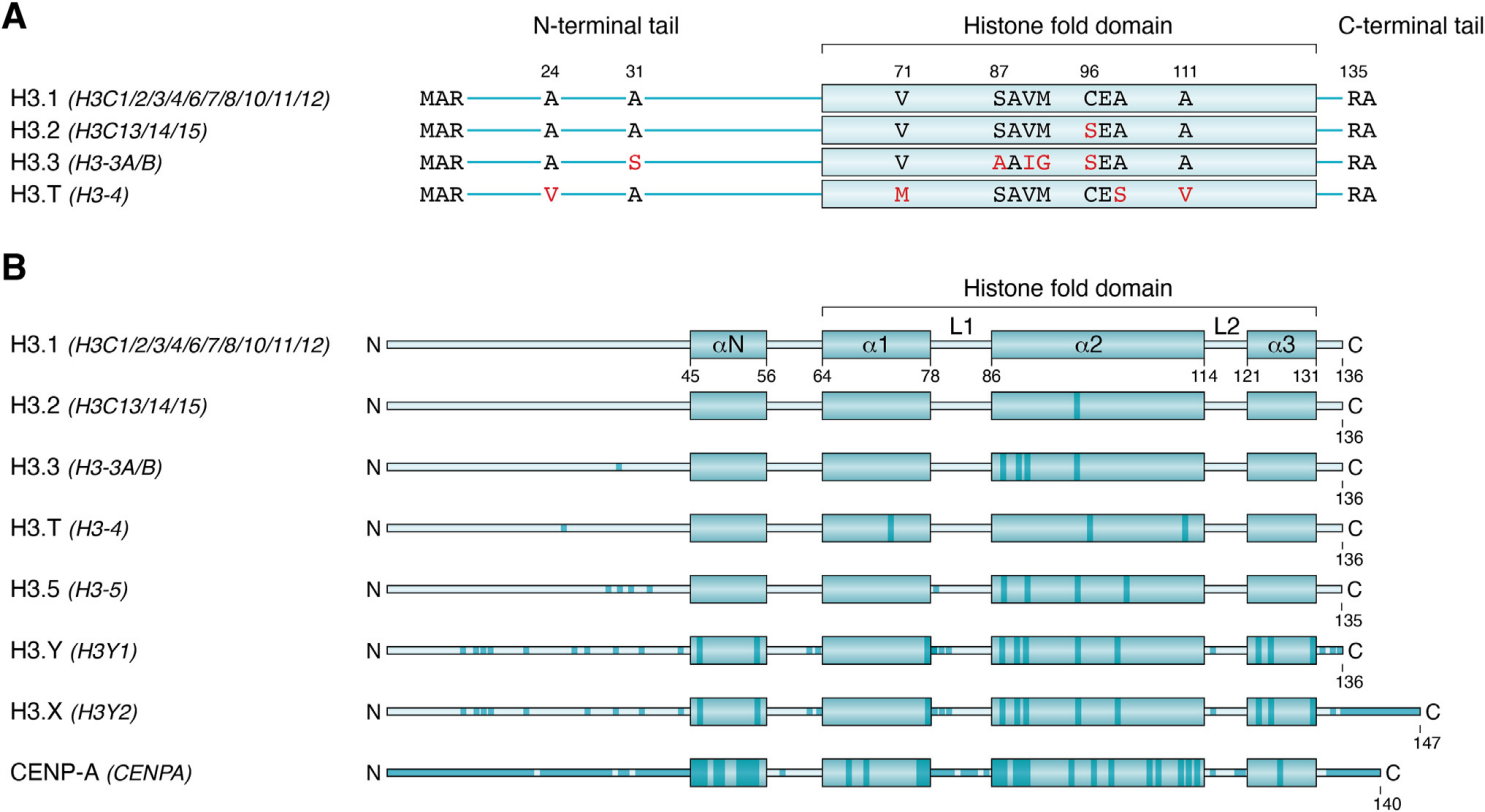
**A schematic representation of H3 variant sequence alignment relative to the canonical H3.***A*, amino acid sequence alignment of histone H3 variants with minor differences compared with the canonical H3 sequence. Sequence alignment was performed using the “UniProt Align” tool. The name of the gene(s) encoding each protein is provided between *parentheses*. The isoform used for the reference H3 sequence is H3.1 (UniProt ID: P68431), encoded by *H3C1/2/3/4/6/7/8/10/11/12* genes. *B*, a schematic representation of amino acid sequence alignment of histone H3 variants with major differences compared with the canonical H3 sequence (UniProt ID: P68431), which is encoded by *H3C1/2/3/4/6/7/8/10/11/12* genes. *Darker-color regions* represent substitutions.

The human histone H3 family contains numerous isoforms. Canonical H3 histones include H3.1 (encoded by *H3C1/2/3/4/6/7/8/10/11/12*) and H3.2 (encoded by *H3C13/14/15*). H3 variants include H3.3 (encoded by *H3-3A* and *H3-3B*), H3.T (also known as H3.4 and encoded by *H3-4*), H3.5 (encoded by *H3-5*), H3.Y (encoded by *H3Y1*), H3.X (encoded by *H3Y2*), and CENP-A (encoded by *CENPA*) (Table 1) (30, 118, 119, 120, 121, 122, 123, 124). Except for CENP-A, H3.Y, and H3.X, most H3 variants have one or just a few amino acid substitutions within the HFD. However, H3 histones are subject to extensive PTMs, so even minor changes in amino acid composition can significantly alter the PTM profile (reviewed in Refs. (125, 126)). For example, H3.2 differs from H3.1 at only one amino acid position (Fig. 4, *A* and *B*) (18), yet S97 substitution in H3.2 occurs at the H3–H4 tetramer accessible surface, which makes it a possible interaction site for H3.2-specific chaperones (127). A recent study using immunoprecipitation experiments discovered that H3.2 also has distinct interaction partners and specific deposition complexes from that of H3.1 (36). H3.1 also has more K14ac than H3.2, a marker associated with gene activation. On the other hand, H3.2 is enriched in K27me2 and K27me3, which are markers associated with gene silencing (119, 128).

#### H3.3

Histone H3.3 is one of the most studied histone variants (63, 118, 129, 130, 131, 132, 133, 134, 135). It differs from canonical H3.1 at five residues (A31S, S87A, V89I, M90G, and C96S) that are involved in histone–histone interactions (Fig. 4, *A* and *B*) (119, 130). In addition, unlike canonical histone H3 genes, H3.3 transcript contains introns. A recent finding demonstrated that nucleosomes containing the H3.3 variant are less stable than nucleosomes containing canonical H3.1 as they are more susceptible to disruption caused by salt, leading to the disassociation of their H2A–H2B dimers (64). Incorporating H3.3 into chromatin influenced its structural organization, leading to a more “open” conformation, and it associates with regions of active transcription (63). Chromatin regions enriched in H3.3 are also generally depleted of H1 (136). The region surrounding amino acids 87 to 90 plays a vital role in chaperone-dependent histone incorporation onto chromatin since it is a specific recognition site for unique chaperones (33, 34). For example, the deposition of H3.3 is facilitated by a particular chaperone called the histone regulator A complex at actively transcribing gene regions (120). In contrast, canonical H3.1 and H3.2 are deposited by a complex called chromatin assembly complex one (CAF1) (118, 137). In addition, the PTMs of H3.3 differ from those of H3.1. For instance, the phosphorylation of H3.3 S31 regulates the accessibility of the heterochromatin area at telomeres by controlling the lysine-specific demethylase enzyme 4B (KDM4B) activity at H3K9 and H3K36 residues (35).

#### H3.T (H3.4)

H3.T differs from H3.1 at four amino acids (A24V, V71M, A98S, and A111V) (Fig. 4, *A* and *B*). *In vitro*, the (H3.T–H4)_2_ tetramer can interact with the H2A–H2B dimers to form a stable nucleosome. Still, histone chaperone Nap1 cannot efficiently incorporate the variant because it cannot dissociate from the histone complex after nucleosome assembly (30). The human Nap2, on the other hand, enhances the deposition of H3.T into nucleosomes and protects the wrapped DNA from enzymatic digestion by micrococcal nuclease (MNase) digestion assay. However, utilizing the Nap1 chaperone in the nucleosome reconstitution stage resulted in more susceptible nucleosome DNA to MNase digestion. Finally, mutagenesis experiments revealed that the V111 plays a vital role in the hNap2-mediated preferential assembly of H3.T-containing nucleosome (30).

#### H3.5

Histone H3.5 differs from H3.1 at nine different positions (Fig. 4*B*) (28). Aside from the missing lysine residue at position 37 in H3.5, T29, S31, C33, N78, A85, G88, S94, and L103 residues of H3.5 correspond to the A29, A31, G33, K79, S86, M89, C95, and F104 residues of H3.1, respectively.

While H3.5 can form stable nucleosome structures, salt-titration experiments revealed that H3.5-containing nucleosomes are only stable at lower salt concentrations than H3.1- and H3.3-containing nucleosomes (28, 123, 138). F104 in H3.1 is located at the interface of H3.1 and H4, forming hydrophobic interactions with the side chains of I34, I50, and T54 residues on H4 (28). Analysis of crystal structure resolutions shows that the substitution of F104 by L103 in H3.5 reduced the hydrophobic interactions between H3.5 and H4, suggesting that this structural difference may account for the instability of the H3.5 nucleosome (28). In the same study, mutational analyses revealed that the H3.5-specific L103 residue is responsible for the instability of the H3.5 nucleosome *in vitro*. In live cells, H3.5 mobility is remarkably faster than for the histone H3.3 variant, suggesting that H3.5 exchanges more rapidly than H3.3. Finally, H3.5 accumulates around TSSs (28), which indicates that H3.5 might regulate access to gene promoters.

#### H3.Y

H3.Y has 30 amino acid substitutions relative to H3.1 (Fig. 4*B*) (139). The side chains of residues K115 and K122 in H3.1 are involved in forming hydrogen bonds with the DNA backbone near the nucleosome entry–exit site (140); these residues are substituted with R115 and R122 in H3.Y. Given that these residues are directly interacting with DNA, the binding between DNA and H3.Y-containing nucleosome might differ from that of H3.1. Indeed, crystal structure revealed that the R42 side chain on H3.1 directly binds to DNA, whereas H3.Y R42 has no contact with DNA binding (124, 141). MNase digestion assay showed that the H3.Y nucleosome was more flexible than other H3 variants containing nucleosomes, as the nucleosomal DNA at the entry–exit site was more accessible to the MNase digestion enzyme (124). In addition, this study shows that the linker histone H1 binds less efficiently to the H3.Y nucleosome and that the DNA end flexibility of the H3.Y nucleosome does translate into higher order chromatin configuration consisting of 12 tandem nucleosome repeats (124). As with H3.5, genome-wide studies show that H3.Y stably assembles at TSSs, promoting an accessible chromatin structure with flexible DNA ends and reduced linker histone H1 binding (124).

#### H3.X

The H3.X amino acid sequence is highly like H3.Y. However, they differ from canonical H3.1 and H3.2 at several key residues, and H3.X has an unusually long C-terminal tail (Fig. 4*B*). These alterations are known to have functional implications. For instance, H3.1 and H3.2 are phosphorylated at S10 and S28 during mitosis and in response to cellular stress (142, 143), but H3.X instead has an A10 and R28 (139). In addition, H3.1, H3.2, and other H3 variants are acetylated at K14 and methylated at K79 (marking actively transcribed regions) (144, 145, 146, 147), but in H3.X, these residues are substituted with Q14 and S79 (139).

#### CENP-A

Centromeric protein A (CENP-A or CenH3) is a histone H3 variant associated with centromeres. It epigenetically marks centromeres and provides a foundation for kinetochore formation (148, 149). It is, therefore, critical for chromosomal segregation and maintenance of genome stability during cell division. CENP-A was first discovered in 1985 as a 17-KDa centromere-associated antigen in CREST (Calcinosis, Raynaud's phenomenon, Esophageal dysmotility, Sclerodactyly, and Telangiectasia) syndrome patients (150, 151). Shortly after this, CENP-A was found to cofractionate with histones during histone purification from nucleosome core particles (152, 153). Later in the 1990s, the complete sequence of the protein was revealed, showing similarity to the canonical histone H3 in amino acid sequence, which led, together with functional analysis of CENP-A expression, to the identification of CENP-A as a histone H3 variant serving as a centromere-specific component of nucleosomal core histones (154, 155).

CENP-A has a typical HFD flanked by N- and C-terminal tails but only shares ∼50% identity with canonical H3.1 or H3.2 (Fig. 4*B*) (156, 157). The CENP-A C-terminal tail retains a hydrophobic region that is crucial for CENP-A’s interaction with the constitutive centromere-associated network (CCAN) protein, CENP-C (148). CENP-A is the only centromere-specific histone variant (Fig. 1*B*), with unique structural features that generate more relaxed nucleosomes and transient DNA unwrapping at the nucleosome entry–exit sites (156, 157, 158, 159, 160, 161). Only 121 bp of DNA wraps around CENP-A-containing nucleosomes (160, 162, 163), most likely caused by the K49 residue at the DNA contact site, corresponding to an R residue in the canonical H3 (27, 164).

CENP-A has fewer PTM sites than canonical H3 (148) but is phosphorylated at S7, S16, and S18 (148, 165, 166). CENP-A S7 is phosphorylated at the beginning of mitosis and dephosphorylated at the end (165, 167). S7-phosphorylated CENP-A is a target for the phosphoserine/threonine-binding proteins 14–3–3 that links phosphorylated CENP-A to CENP-C, allowing for the efficient formation and maintenance of active kinetochore (165). CENP-A S16 and S18 phosphorylation is cell cycle independent and essential for chromosome segregation during mitosis (166). This limited profile of PTMs seen in CENP-A may reflect its restricted and time-specific role in mitosis, in contrast to the canonical histone H3, which has a wide range of implications in gene regulation (148). CENP-A also has a 2-amino acid insertion (R80, G81) within the HFD, which mediates the interaction with the constitutive CCAN protein CENP-N (168). CENP-A is chaperoned to the centromere by Holliday junction recognition protein and is associated with 16 known CCAN proteins, including CENP-C and CENP-N (27).

### H4 variants

**Figure 5:**
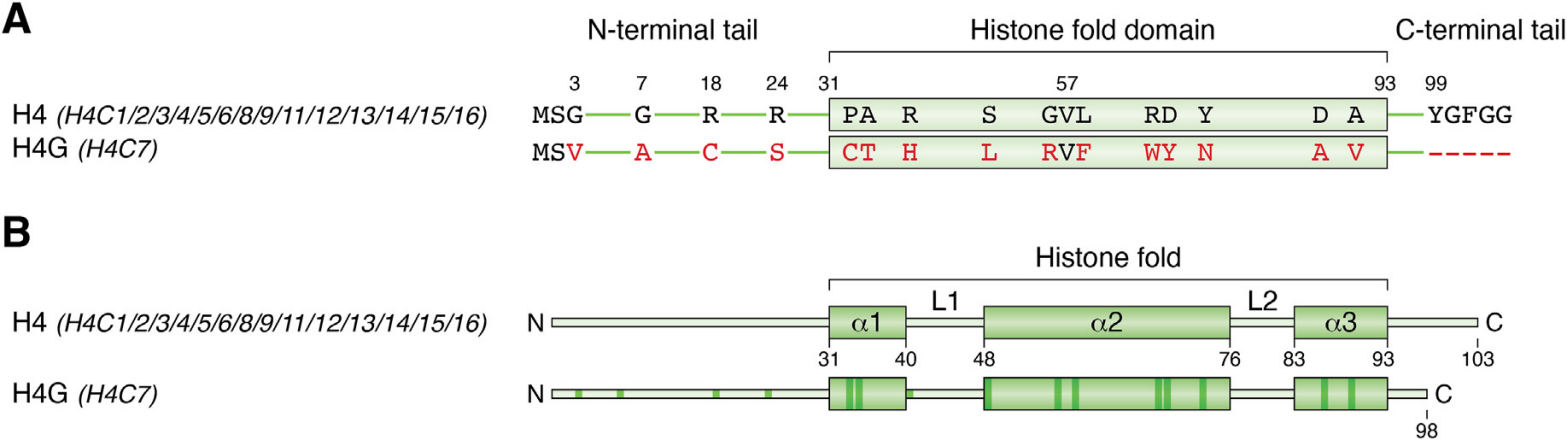
**A schematic representation of H4 variant sequence alignment relative to the canonical H4.***A*, amino acid sequence alignment of histone H4 variant compared with the canonical H4 sequence. Sequence alignment was performed using the “UniProt Align” tool. The name of the gene(s) encoding each protein is provided between parentheses. The isoform used for the reference H4 sequence is the product of *H4C1/2/3/4/5/6/8/9/11/12/13/14/15/16* genes (UniProt ID: P62805). *B*, a schematic representation of the amino acid sequence alignment of the histone H4 variant compared with the canonical H4 sequence, with *darker-colored regions* representing substitutions.

H4 exhibits the most conserved amino acid sequence among all histone families, and H4.G (H4.7) is the only known H4 variant. H4.G is encoded by the *H4C7* gene in histone cluster one on chromosome six (169). H4.G has 85% amino acid identity with canonical H4 but is truncated by five amino acids in the C-terminal tail (Fig. 5, *A* and *B*). H4.G amino acid sequence differences occur at important H2A, H3–H4 tetramer, and the H2A–H2B dimer contact sites and may explain why H4.G cannot form stable nucleosomes *in vitro* (170). These include the C-terminal residues essential for cell viability in yeast (171).

## Histone variants and their role in disease

**Figure 6:**
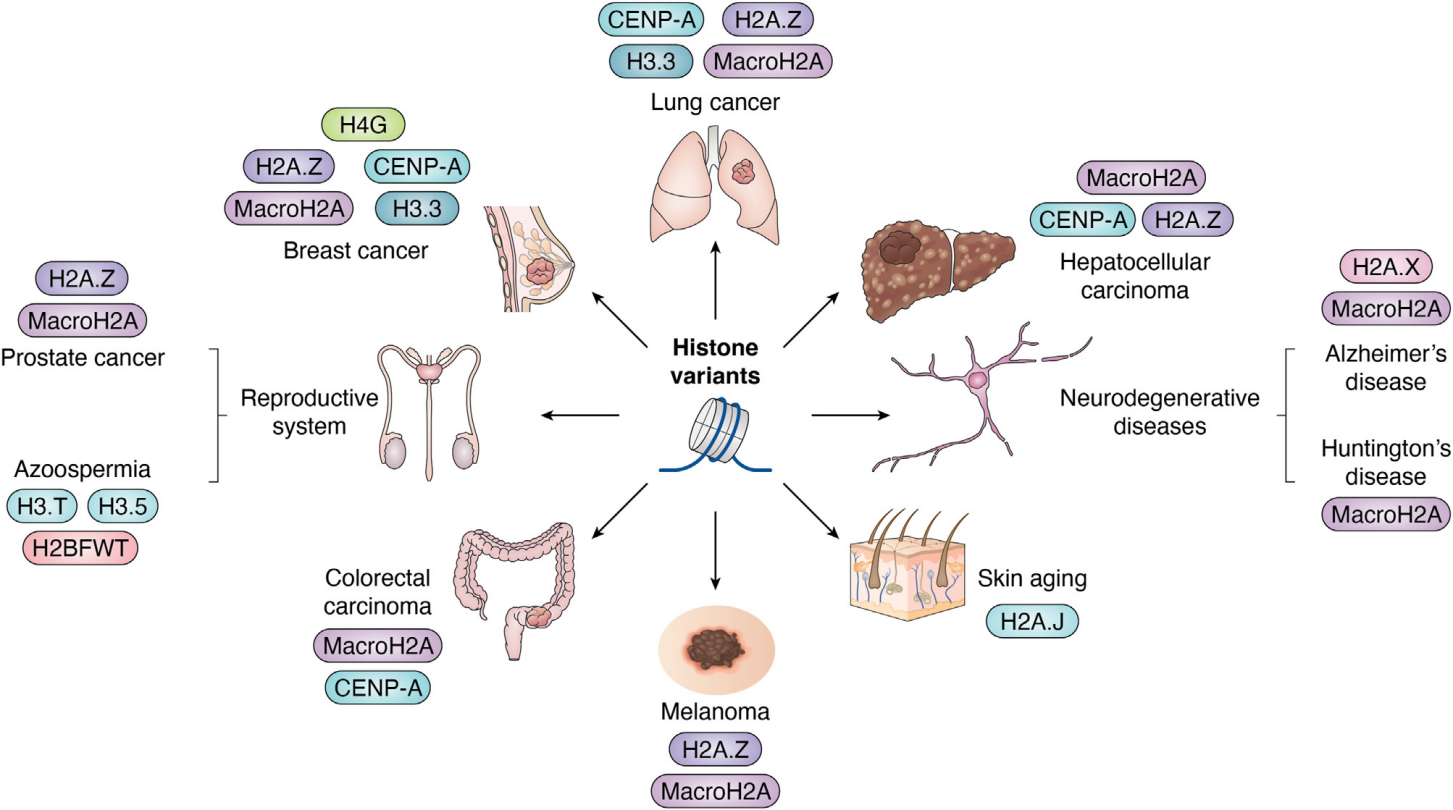
**Involvement of histone variants in human diseases.** This figure shows some examples of the association of various histone variants with different human diseases. The diseases depicted include cancer, reproductive disorders, and neurodegenerative diseases, illustrating the critical impact of histone variant dysregulation on human health. Of note, this figure highlights examples of the “discovered-to-date” disease association of histone variants, and it does not imply that certain variants are exclusively associated with specific diseases.

Because histone variants alter nucleosome and chromatin structures, it should be no surprise that histone dysregulation and mutations alter gene expression patterns that either cause or propagate disease. Here, we provide a few examples of histone variants linked to human diseases, such as cancer and neurological disorders (Fig. 6).

### H2A variants

H2A.Z genomic occupancy at the TSS modulates the plasticity of embryonic stem cells and can activate or repress epithelial-to-mesenchymal transition genes (172). In tumors, H2A.Z overexpression correlates with increased rates of proliferation, metastasis, and resistance to conventional therapies (173, 174, 175). H2A.Z hypoacetylation also silences tumor suppressor genes in prostate cancer (176). Among H2A variants, H2A.Z.2, H2A.X, and macroH2A.1.2 are crucial in defining the molecular signatures of aneuploidy and chromosomal instability across diverse cancers (39, 177, 178). Since H2A.Z is involved in DNA repair, its depletion has been shown to lead to DNA damage, oxidative stress, and premature muscle aging in mice (179).

Studies have found that H2A.X and macroH2A are commonly downregulated in cancers, whereas H2A.Z is frequently upregulated (25, 39). In mice, loss of H2A.X is associated with increased chromosomal aberrations, genome instability, and susceptibility to lymphomagenesis, a phenotype significantly accelerated in the absence of p53 (180, 181). Interestingly, hypoxic conditions increase the γ-H2A.X/H2A.X ratio and promote angiogenesis in hepatocellular carcinoma (182), indicating that external cues can modulate the occupancy of H2A variants. H2A.X is an emerging cancer biomarker since its levels are indicative of tumor size/progression and patients’ responsiveness to therapy (25, 183). Practically, measuring γ-H2AX levels in peripheral blood cells postirradiation has shown clinical relevance in assessing bladder and colorectal cancer risk and development (184, 185, 186). Thus, this approach highlights the importance of PTMs of histone variants as promising biomarkers and potential targets in cancer research.

Moreover, low macroH2A levels induce chromatin plasticity, stem-like characteristics, and oncogenic signaling pathways in bladder and prostate cancers (187, 188). While some H2A variants have definitive functions in cancer (either antioncogenic or pro-oncogenic), macroH2A exhibits dual roles. Specifically, splicing factors and DNA modifications also trigger pro-oncogenic properties of macroH2A (reviewed in Refs. (189, 190)). Studies in mice have also shown that depleting macroH2A is associated with retarded growth and reproduction and metabolic dysfunction (191).

Studies from human fibroblasts demonstrated that histone variant H2A.J accumulation enhances the signaling of senescent cells to the immune system and may play a role in chronic inflammation and the development of aging-associated diseases (192, 193).

Abnormal H2A.B levels modulate the chromatin and impact alternative splicing patterns (194). In Hodgkin lymphoma, H2A.B overexpression shortens the S-phase and impairs the DNA damage and repair process (87). Interestingly, H2A.B contains amino acid residues comparable to the oncogenic mutations frequently observed in canonical histone H2A, as if H2A.B is, in fact, a “mutated” H2A or a “ready-made” oncohistone (25, 194). Thus, H2A.B dysregulation may relieve the repressive state of oncogenic factors, yielding a neoplastic phenotype, probably through the relaxed and unstable structural features of H2A.B–nucleosomes.

### H2B variants

TSH2B is a testis-specific H2B variant crucial for the histone–protamine transition during spermatogenesis and is linked to male fertility in humans (113, 195) and mice. TSH2B-mutant mice in which the C-terminal domain was modified were infertile (114). However, TSH2B-null mice were fertile, suggesting a compensatory mechanism for rescuing the TSH2B deficiency (114, 196, 197). H2BFWT, the H2B.W subtype, is also testis specific. SNPs in this gene were found to be highly linked to male infertility (113, 195, 198, 199, 200). An E76K mutation in the HIST1 cluster H2B HFD interferes with H2A–H2B interaction and distorts the interface between H2B and H4 (201, 202). This mutation enhances colony formation and cellular proliferation *in vitro* and correlates with bladder, head, and neck cancers (201, 202). A G53D mutation occurring mainly in HIST2 cluster H2B variants disrupts histone–DNA interaction, enhances *in vitro* cell migration, and correlates with pancreatic ductal adenocarcinoma (202, 203).

### H3 variants

Interestingly, H3 variants are differentially expressed across tissues, which implies they are involved in cell differentiation and tissue development. For instance, H3.T and H3.5 variants are highly expressed in the testes, and their expression is crucial for early spermatogenesis and proper entry into meiosis (18, 28, 204). Dysregulated H3.T and H3.5 expression can lead to azoospermia (138, 196). In muscles, H3.3 deposition plays essential roles in the various stages of muscle cell differentiation from mesodermal precursors, especially at the *MYOD* gene promoter region in myotubes (205, 206, 207). H3.3 is also highly expressed during embryonic gastrulation, and depleted H3.3 levels arrest gastrulation and reduce the anteroposterior axis, hindering further development (208, 209). H3.3 mutations have been identified in developmental disorders, giant cell glioblastoma (a rare brain tumor), chondroblastoma, and giant cell tumors of the bone (210, 211, 212). An H3.3 G34R/V mutation is linked to pediatric gliomas and significantly hinders brain development, impairs neurocognitive functions, and contributes to the development of other central nervous system tumors (213, 214). This mutation also alters chromatin dynamics and promotes tumor growth by dysregulating gene expression related to cell proliferation and differentiation (215). An H3.3 K27M is involved in diffuse midline glioma and increased tumor cell resistance to ionizing radiation therapy (216, 217). Finally, H3.3 alterations are implicated in aging-related processes and cellular senescence, where chromatin structure maintenance is crucial for maintaining genomic stability and cellular function over time (218, 219).

### H4 variants

Histone H4 mutations are rare (10), and little information exists about H4 variants and disease. H4.G is overexpressed depending on the breast cancer tumor stage and enhances rDNA transcription (170) by making chromatin more accessible (220).

## Concluding remarks and future directions

Significant progress has been made in deciphering how histone variants are incorporated into chromatin and alter nucleosome and chromatin structures. Histone variants contribute to many cellular processes, physiology, and disease (Figs. 1*B* and 6). They play a pivotal role in transcription regulation. For example, H2A.Z, H2A.B, and H3.5 are primarily associated with transcriptional activation, whereas MacroH2A is mainly associated with gene repression. Some histone variants are essential for DNA repair (H2A.X) and DNA replication (H2A.Z and CENP-A) and are associated with telomeres (H3.3 and macroH2A) (Fig. 1*B*).

The late David Allis (founder of the histone code) famously said that "every amino acid in a histone matters" (221), and histone variant research to date reiterates this fundamental point. Substitutions in the amino acid sequence of histones can alter PTMs; histone–histone interactions within the nucleosome particle or with the linker histone; histone–DNA interactions; or interactions with specific chaperones, chromatin remodelers, readers, writers, and erasers, all of which influence chromatin structure and gene expression. Nevertheless, more work is needed to decode crosstalk between histone variants, canonical histones, and PTMs and how histone variants regulate specific gene expression programs.

With continued advances in mass spectrometry instruments and methods (222, 223), we should see improved understanding of the histone variant interactome and the chromatin-associated protein networks. Unraveling the regulatory network of histone variants and their deposition into chromatin is crucial for understanding their precise roles in disease initiation and progression. It could eventually lead to the identification of novel therapeutic targets. Recent advances in structural biology techniques like cryo-EM might help elucidate whether these small amino changes have any consequences on the overall nucleosome structure and how those structural shifts modulate gene expression. These, too, might reveal areas where novel small-molecule inhibitors might help prevent or reverse disease. Because histone variants are associated with fertility, development, cellular differentiation, and many diseases, they may ultimately be used as diagnostic markers. Future research should also focus on identifying the functional roles of the lesser-known histone variants, especially H2B variants. Still, all research focused on histone variant biology will undoubtedly improve our understanding of human diseases and chromatin-associated disorders.

## Conflict of interest

The authors declare that they have no conflicts of interest with the contents of this article.
